# Extraction of first permanent molars severely affected by molar incisor hypomineralisation: a retrospective audit

**DOI:** 10.1007/s40368-021-00647-w

**Published:** 2021-06-25

**Authors:** I. J. Brusevold, K. Kleivene, B. Grimsøen, A. B. Skaare

**Affiliations:** Department of Paediatric Dentistry and Behavioural Science, Institute of Clinical Dentistry, University of Oslo, Oslo, Norway

**Keywords:** Molar incisor hypomineralisation, Extraction, Paediatric dentistry

## Abstract

**Aim:**

The aim of this study was to evaluate possible spontaneous space closure after extraction of first permanent molars in children and their eventual need for orthodontic treatment.

**Methods:**

Twenty-seven children with at least one first permanent molar planned for extraction were enrolled in the study. The children were referred to the Department of Paediatric Dentistry, University of Oslo, between 2009 and 2017. All extracted teeth were severely affected by Molar Incisor Hypomineralisation and/or caries. The children and their parents had consented to extraction and follow-up. Data were analysed with SPSS 26.

**Results:**

The age of the children was between 5.5 and 12.1 years (mean 8.7) at extraction. The mean follow-up time was 3.2 years (range 1.1–6.3). Sixteen children (59.3%) had all four molars extracted, five (18.5%) had three, five had two and one had one molar extracted. In the maxilla, the second permanent molar had erupted in the place of the first molar in all the children, and none of them needed orthodontic space closure. In the mandible, eight children (29.6%) needed orthodontic treatment to close the spaces after extraction. In three children, the second molar was not yet erupted and treatment need was not settled.

**Conclusion:**

Extraction of severely affected first permanent molars before the eruption of the second molar is a treatment option causing little additional treatment in the majority of cases.

## Introduction

The treatment of severely hypomineralised permanent molars in children is a challenge. Concerns has to be taken to age, symptoms, compliance, lifelong treatment need and the opinion of parents. A common diagnosis related to dental hypomineralisation, with a mean global prevalence of 13%, is “Molar Incisor Hypomineralisation”, commonly referred to as MIH (Schwendicke et al. 2018). MIH is primarily affecting first permanent molars (FPM) and permanent incisors, although similar defects can be found in permanent canines, premolars and second primary and permanent molars (Weerheijm et al. 2003; Schmalfuss et al. 2016; Elfrink et al. 2012; Kevrekidou et al. 2020).

Hypomineralisation is the result of a disruption during the maturation phase of amelogenesis (Varga et al. 2015). The hydroxyapatite matrix is produced in the normal thickness, while the uptake of minerals and the degradation of the protein matrix is being disrupted. This results in an enamel with lower mineral content and a corresponding higher protein content (Mangum et al. 2010). The reduced mineral content results in a structure that alters the reflection of light, giving an opaque area on the enamel. In addition, the protein present might be discoloured, resulting in opaque white chalky, yellow or brown spots (Elhennawy et al. 2017b). In MIH, these discoloured spots are demarcated, in contrast to the more diffuse spots seen in dental fluorosis. The hypomineralised areas have a lower surface strength, and post-eruptive breakdown is frequently observed (Kramer et al. 2018). The fact that the enamel is more porous, can also lead to bacterial invasion and pulpal inflammation with increased amounts of cytokines such as TRPV-1 with a corresponding hypersensitivity to temperature (Rodd et al. 2007; Fagrell et al. 2008). In addition, the inflammation-associated lowered pulpal pH can reduce the effect of local anaesthetic drugs (Becker, Reed 2012). These factors contribute to making restorative treatment painful to the child, which in turn increases the risk of dental fear and anxiety and can give treatment fatigue.

It has been reported that children with MIH undergo ten times more treatment than children without MIH (Jalevik, Klingberg 2002). Therefore, for the most severely affected molars, extraction might be a good alternative, suggested in several guidance and guideline documents (Lygidakis et al. 2010; Cobourne et al. 2014; Ashley, Noar 2019). However, extraction has been considered a poor alternative by some orthodontists. It will often cause orthodontic treatment need, or longer treatment periods when treating malocclusion, as a missing first permanent molar gives less effective anchorage for orthodontic forces (Williams, Gowans 2003; Ong, Bleakley 2010). Other orthodontists are more positive to extractions, and have contributed to clinical guidelines in Great Britain (Cobourne et al. 2014). Previously, extraction of FPM was common due to caries. The rationale was dual; extraction would remove heavy treatment need of the tooth in question and it was believed that extraction of a tooth with active caries would have a caries-preventive effect (Thilander, Skagius 1970). The need for extraction because of caries has decreased in the last decades. The rationale for extracting molars seriously affected by MIH is no longer caries prevention for the rest of the dentition, but rather consideration of the long-term prognosis for the tooth in question. In addition, the total burden of treatment and symptoms for the child is considered (Ashley, Noar 2019; Patel et al. 2017; Lygidakis et al. 2010). The ideal age for extraction has been considered to be between 8 and 10 years or with the tooth in Demirjian stage E (Thunold 1970; Teo et al. 2013). However, sometimes the child’s symptoms and estimated treatment burden lead to a need for treatment at an earlier age. In other cases, extraction was not considered as the “ideal”, but emerged as the best option later.

Very few follow-up studies evaluating spontaneous space closure after extraction of FPM exist. Therefore, the aim of this study was to evaluate spontaneous space closure after extraction of 1–4 FPM severely affected by MIH without strictly considering optimal age at extraction.

## Materials and methods

This study a is retrospective audit following children referred to the Department of Paediatric Dentistry, University of Oslo for extraction of FPM between April 2009 and January 2017. The children were re-examined in 2019 after the eruption of their second permanent molars. At follow-up, the level of space closure after extraction was examined.

Before extraction, the children were examined clinically and by panoramic radiographs by an experienced dentist under the surveillance of a specialist in paediatric dentistry. An orthodontist was consulted when necessary. Extraction was not recommended when there were missing permanent teeth and in cases with severe malocclusion where extraction of FPMs would complicate orthodontic treatment. All the extracted teeth were severely affected by MIH. The treatment options for the affected teeth were considered to be temporary restorations with stainless steel crowns or large fillings for later indirect restorations, or extraction. The treatment options were discussed with the parents. Thus, the children included in this study were those who chose extraction after being presented with the above-mentioned alternatives and consented to extraction and follow-up either at the Department of Paediatric Dentistry or at the referring clinic in the Public Dental Service (PDS). All children in Norway are recalled for a dental check-up at one to 2-year intervals. At follow-up in the PDS, the presence and position of second permanent molars (SPM) were examined on radiographs and with clinical examination. Radiographs were then sent to the Department of Paediatric Dentistry and examined by a paediatric dentist. At follow-up at the Department of Paediatric Dentistry, the children were examined by a paediatric dentist that evaluated the space between the 2nd premolar and the 2nd molar and the tilting of the 2nd molar both clinically and on radiographs. At follow-up, both in the PDS and the Department of Paediatric Dentistry, the decision whether to refer to orthodontic treatment was based on clinical discretion and discussion with the child and parent. However, the final decision of starting orthodontic treatment after the referral was taken by the orthodontist and the family.

All radiographs, and clinical photos when available, were evaluated by a specialist in pediatric dentistry. The results were considered “good” when the SPM was in the place of the FPM without spaces. A small space with a slight tilting of the second permanent molar was considered “acceptable”. Larger spaces and tilting were considered “not acceptable”. Space closure of the upper and lower jaws were evaluated separately.

### Statistical analyses

Data were analysed with SPSS v 26 (IBM SPSS Statistics). Frequencies and means were explored with descriptive statistics.

### Ethical considerations

The project was considered a quality assurance project and approved by the Norwegian Data Protection Services (project number 56467).

## Results

**Fig. 1:**
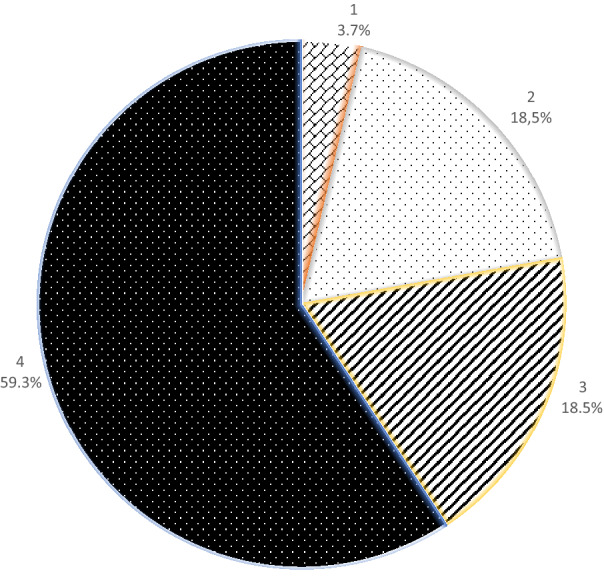
Pie chart illustrating the number of teeth extracted per person. A large majority (16/59.3%) of children had all four FPM extracted. Five children (18.5%) had three FPM extracted, another 18.5% had two while one (3.7%) had one FPM extracted

**Fig. 2:**
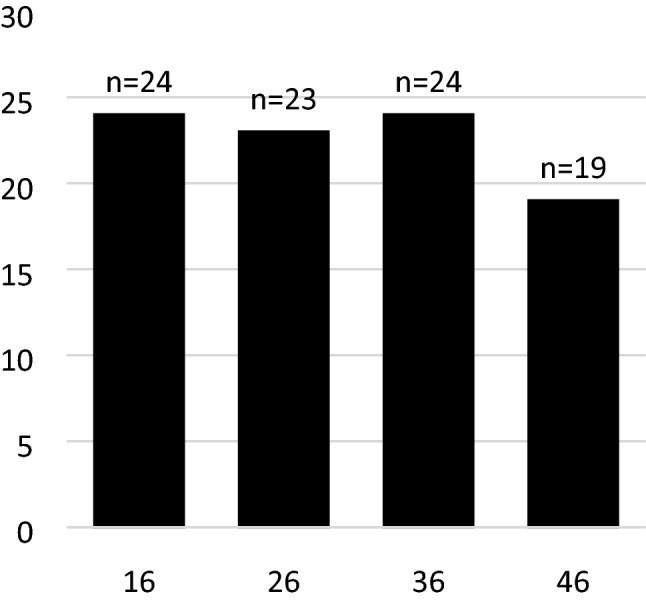
Number of extracted molars. Four bars representing four first permanent molars. *N* = number of first molars extracted per quadrant. In total, 90 teeth were extracted in 27 children

Most of the children (*n* = 16) had all four FPMs extracted. Five children had three, five had two and one had one FPMs extracted (Fig. 1). The mean number of extracted teeth per child was 3.3. The upper right and the lower left FPM were the most frequently extracted teeth as they were extracted in 24 (88.9%) of the children. The lower right was the least frequently extracted, in 19 (70.4%) of the children (Fig. 2). Altogether 90 teeth were extracted in 27 children. The mean age at extraction was 8.7 years with a range from 5.5 to 12.1 years. The mean follow-up time was 3.5 years.

### Space closure

Twenty-four children had one or two maxillary FPM extractions. Twenty-two children experienced full space closure at follow up. In one child the maxillary FPM was not fully erupted but showed a favourable direction as seen on radiograph. This means that the SPM drifted mesially and erupted in the place of the FPM. In one child there was a small space that was considered acceptable without treatment need.

**Fig. 3:**
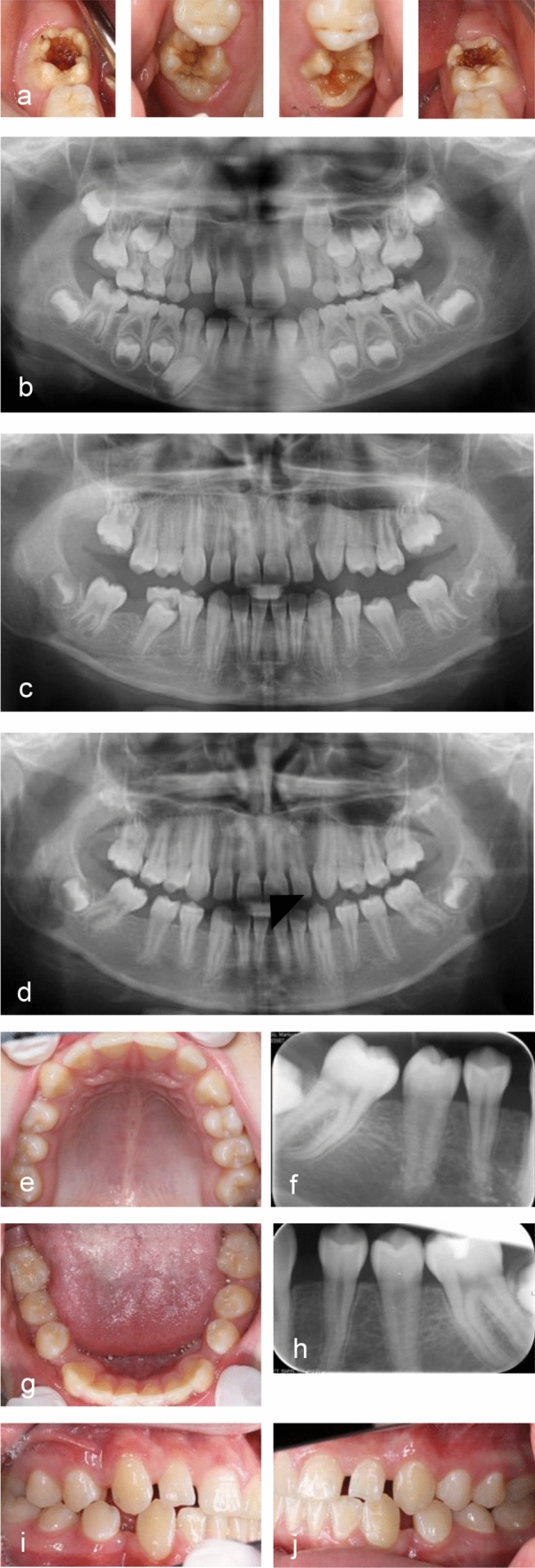
Picture series of child having all FPM extracted at 9.5 years age. **a** Clinical photographs before treatment show that all FPM have severe decay. The child experienced pain from the molars. **b** Panoramic x-ray at 6 years. Ectopically erupting upper FPM. Posteruptive breakdown and caries. The orthodontist did not recommend extraction and the teeth were treated with semipermanent fillings until extraction. **c** Panoramic x-ray at 11 years. **d** Panoramic x-ray at 13 years. Full space closure is seen in the maxilla. In the mandibula, there are spaces on both sides and tipping of tooth 47. **e–j** At age 15, the child has no subjective treatment need and is happy with his teeth. Spaces are seen on both sides. The tipping of 47 is somewhat reduced since the previous control and 48 is present. **e, g**: Clinical photographs, occlusal views. **f**, **h** Apical radiograph from 4th and 3rd quadrant. **i**, **j** Clinical photographs buccal view both sides

**Fig. 4:**
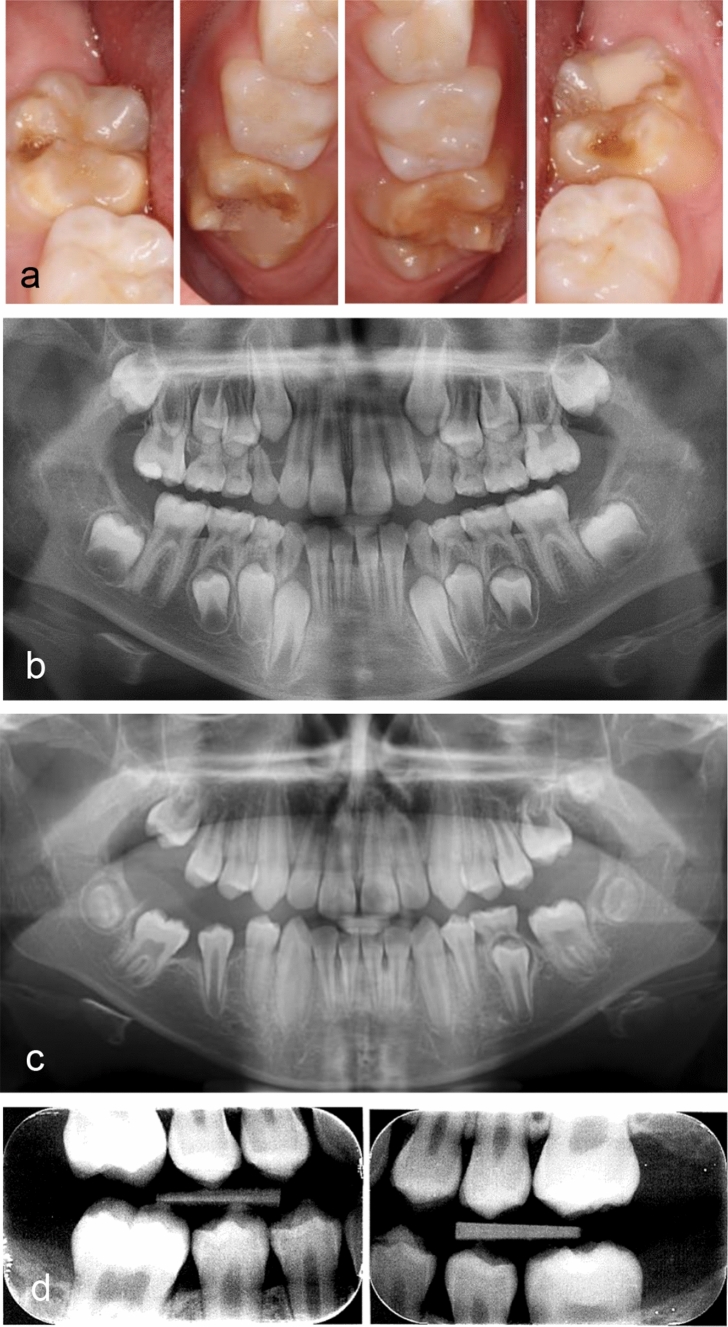
Good results after extraction of all FPM. **a** Clinical photographs before extraction at 8.5 years. Hypomineralised enamel are seen on all surfaces. Fillings have been placed after enamel breakdown in 16 and 36. The child experienced pain and hypersensitivity from all FPM and the child and parents preferred extractions instead of life-long restorative treatment need. **b** Panoramic radiograph before extraction at 8.5 years. **c** Panoramic radiograph at follow-up 1.7 years later when the child was 10.2 years. **d** Bitewing radiographs at 11 years show stable occlusion without spaces or tipping of second permanent molars

Twenty-five children had one or two mandibular FPM extractions. In the lower jaw, full space closure was seen in six children, while there was a space considered acceptable in another eight children. A larger space or tilting of the SPM was observed in eight children. In three of the children, the mandibular SPM was not yet fully erupted and treatment need could not be determined, however, based on radiographic evaluation, they had a favourable direction of eruption. Thus, sixteen of the children (59.3%) had no need for orthodontic space closure because of the FPM extractions, while eight of them did. Figures 3 and 4 show examples before extractions and at follow-ups.

Overall, eight children had good results, eight children had acceptable results and eight children had not acceptable results. When judging the upper jaw separately, 22 children had good results and one child had an acceptable result. For the lower jaw, six children had good results, eight children had acceptable results and eight children had not acceptable results.

## Discussion

In this study, we assessed the level of spontaneous space closure after FPM extractions. Some of these children had mandibular spaces and tiltings of SPMs requiring orthodontic treatment. According to a report from Statistics Norway, approximately 50% of Norwegian children receive orthodontic treatment for various reasons (Ekornrud et al. 2019). However, orthodontic treatment is easily available in Norway. Even though treatment is costly, there is quite generous economic support through the national social insurance system. In Norway, all dental treatment except orthodontic treatment is free of charge for children up to eighteen years of age. Therefore, some families would prefer restorative treatment before extraction if they know that orthodontic treatment will be needed. However, repeated restorative treatment will also lead to economic cost beyond the age of eighteen, therefore orthodontic treatment cost is not often decisive. In a German study, it was found that extraction and orthodontic alignment could be cost-effective compared to restoration in the German system, especially where more than one tooth was affected (Elhennawy et al. 2017a).

We found that maxillary extractions resulted in spontaneous space closure for all the included children (age range 5.5–12.1 years). Therefore, none of the children would need orthodontic treatment because of maxillary FPM extractions. This is in line with previous findings by Thunold (1970), who reported spontaneous maxillary space closure in all patients in a 25-year follow-up study. The study by Thunold included 52 individuals with the early loss of FPMs and no orthodontic treatment. In addition to spontaneous space closure, the individuals had less anterior maxillary crowding than two other populations with no loss of FPM. Two other studies also reported favourable results for the maxilla (Thilander, Skagius 1970; Teo et al. 2013). Therefore, extraction of maxillary FPM before the eruption of the SPM can be recommended as a good treatment option in severe cases of MIH.

The results in the present study were less favourable after mandibular FPM extractions. Fourteen (51.6%) of the children with mandibular extractions had complete or acceptable spontaneous space closure. Eight of the 27 children (29.6%) needed mandibular orthodontic space closure. Our results are comparable with the results reported by Thunold (1970). In that study closure in the lower jaw was assessed as unsatisfactory in 30% of the cases. A more recent Swedish study reported unsatisfactory space closure in 26% of the cases (Jalevik, Moller 2007).

Some dentists are reluctant to extract permanent teeth and advocate restorative and endodontic treatment and it is nearly always possible to save an FPM when the root is not affected (Linner et al. 2020; Bekes 2020). However, our main argument to extract is the lifetime burden of cost and effort for the individual that has to be considered when treatment decisions are made. For many children, an early extraction of their poor and often painful tooth, gives a smaller total burden than repeated restorative treatments. A Swedish study showed that children with MIH received ten times more treatment and had a higher dental fear and anxiety score than their healthy controls (Jalevik, Klingberg 2002).

In the present study, the age of the children at extraction was in the range 5.5–12.1 years. Age was not a determining factor for the future need for orthodontic space closure in our material. Worth mentioning is that the youngest child, who was 5.5 years at the extraction of all four FPMs, experienced full space closure and had no need for orthodontic treatment because of the extractions. The child with the highest age at extraction (12.1 years) experienced full maxillary space closure while mandibular spaces required orthodontic closure. When evaluating the radiographs at follow-up, some of the children who had small spaces and tilting only, had been referred to orthodontic treatment. However, others with similar occlusion and spaces did not experience treatment need (See example, Fig. 3). It is, of course, the children and their families who decide whether they want to receive orthodontic treatment. An important reservation concerning the results in our study is the inclusion of cases. In children with agenesis or unfavourable occlusion at first examination (e.g. large overjets, class III occlusion), extractions could be delayed or avoided after consulting an orthodontist.

## Conclusion

Extraction of FPMs severely affected by MIH can be a treatment option in some cases. For maxillary extractions, spontaneous space closure can be anticipated while mandibular FPM may need orthodontic space closure. Orthodontic evaluation is recommended at the time of the planned or enforced extraction.

## Data Availability

Not applicable.
